# Relevance of hoarding behavior and the traits of developmental disorders among university students: a self-reported assessment study

**DOI:** 10.1186/s13030-019-0156-1

**Published:** 2019-06-03

**Authors:** Kosuke Kajitani, Rikako Tsuchimoto, Jun Nagano, Tomohiro Nakao

**Affiliations:** 1 Center for Health Sciences and Counseling, Kyushu University, 6-1 Kasuga-koen, Kasuga, Fukuoka, 816-8580 Japan; 2 Department of Neuropsychiatry, Graduate School of Medical Sciences, Kyushu University, 3-1-1 Maidashi Higashi-ku, Fukuoka, Japan

**Keywords:** Hoarding behavior, ADHD, Autism spectrum disorder, University students, CIR, ASRS, AQ

## Abstract

**Background:**

Previous studies have shown that hoarding behavior usually starts at a subclinical level in early adolescence and gradually worsens; however, a limited number of studies have examined the prevalence of hoarding behavior and its association with developmental disorders in young adults. The aims of this study were to estimate the prevalence of hoarding behavior and to identify correlations between hoarding behavior and developmental disorder traits in university students.

**Methods:**

The study participants included 801 university students (616 men, 185 women) who completed questionnaires (ASRS: Adult ADHD Self-Report Scale version 1.1, AQ16: Autism-Spectrum Quotient with 16 items, and CIR: Clutter Image Rating).

**Results:**

Among 801 participants, 27 (3.4%) exceeded the CIR cut-off score. Moreover, the participants with hoarding behavior had a significantly higher percentage of ADHD traits compared to participants without hoarding behavior (HB(+) vs HB(−), 40.7% vs 21.7%). In addition, 7.4% of HB(+) participants had autism spectrum disorder (ASD) traits, compared to 4.1% of HB(−) participants. A correlation analysis revealed that the CIR composite score had a stronger correlation with the ASRS inattentive score than with the hyperactivity/impulsivity score (CIR composite vs ASRS IA, *r* = 0.283; CIR composite vs ASRS H/I, *r* = 0.147).

**Conclusions:**

The results showed a high prevalence of ADHD traits in the university students with hoarding behavior. Moreover, we found that the hoarding behavior was more strongly correlated with inattentive symptoms rather than with hyperactivity/impulsivity symptoms. Our results support the concept of a common pathophysiology behind hoarding behavior and ADHD in young adults.

## Background

Hoarding is defined as the acquisition of and failure to discard possessions of little use or value [1]. Hoarding behavior can cause clinically significant distress and impairment in physical, occupational, and social situations. Historically, hoarding has been categorized as a subtype or dimension of obsessive-compulsive disorder (OCD). However, most patients with hoarding behavior do not meet other criteria for OCD, and most patients with OCD do not show significant hoarding behavior [2, 3]. Furthermore, the prevalence of hoarding behavior is actually higher than that of OCD [4]. The idea that hoarding might be an isolated entity has led to the creation of a new disorder, “hoarding disorder,” in the Diagnostic and Statistical Manual of Mental Disorders fifth edition (DSM-5) [5, 6]. Thus, knowledge regarding hoarding behavior is changing.

The prevalence of hoarding behavior in the general population has been reported to be 2–6%, suggesting that hoarding behavior is not rare [4, 7, 8]. Indeed, in addition to the growing body of scientific literature, hoarding has been gathering public attention in the media, including on television [9, 10]. Even in Japan’s media, hoarding behavior is sometimes reported as “*gomi*-*yashiki,*” literally meaning rubbish (*gomi*) house (*yashiki*). Hoarding behavior increases the risk of falls, injuries, fires, and infection, thus threatening the safety and health not only of the hoarder but also of the community. Overall, hoarding behavior is a growing social problem in advanced countries.

The characteristics of hoarding behavior have been examined from the viewpoints of comorbidity and trajectory. For example, major depression and anxiety disorders (including social phobia and generalized anxiety disorder) showed high comorbidity with hoarding behavior in a large-scale study [2]. Another study indicated that lifetime alcohol dependence was significantly more prevalent in participants with clinically significant hoarding behavior compared to a control group (52.2% vs 19.5%) [4]. In addition, hoarding behavior has often been observed in older adults with dementia [11]. In fact, comorbidity occurs in up to 92% of cases meeting the diagnostic criteria for hoarding disorder [2, 12]. This high comorbidity rate with other mental disorders complicates the diagnostic picture of hoarding disorder [13]. With respect to the trajectory, hoarding behavior has been regarded as a chronic and progressive psychiatric condition. Once considered a problem of old age, hoarding symptoms often come to clinical attention when patients are late in life [14]. One investigation found hoarding to be nearly three times as prevalent among older adults than younger adults [4]. Furthermore, some reports have suggested that hoarding symptom severity increases with age [14, 15]. However, retrospective studies indicate that hoarding behavior appears to emerge in childhood or adolescence. For example, some reports show that the median age of onset for hoarding symptoms is 10–20 years [16, 17]. Furthermore, other studies have reported that hoarding behavior usually starts at a subclinical level in early adolescence and gradually worsens [14, 17]. These results suggest that hoarding behavior may require earlier intervention.

Therefore, to understand the pathogenesis and progression of hoarding symptoms, it is useful to examine hoarding behavior from the viewpoint of developmental disorders. Indeed, there has been accumulating evidence that suggests an association between hoarding and developmental disorders. For example, Hacker et al. reported a high prevalence of hoarding in children with attention deficit hyperactivity disorder (ADHD) [18]. In addition, Storch et al. demonstrated that hoarding symptoms were both common and clinically significant in children with autism spectrum disorder (ASD) [19]. Thus, there has been an increase in research that examined the correlates of hoarding among children with developmental disorders; however, few studies have investigated the characteristics of hoarding symptoms in young adults. In particular, there have been no large-scale studies that have examined the correlations between hoarding behavior and developmental disorders in university students. In the present study, we examined the correlations between hoarding behavior and developmental disorder traits among university students using a clutter image rating and self-reported questionnaires for ASD and ADHD.

## Methods

### Study participants

This observational study was conducted at Kyushu University from April 2016 to April 2018. All participants were students who volunteered to participate and were recruited through the University’s Interdisciplinary Graduate School of Engineering Sciences and Faculty of Arts and Science. We explained the study outline and asked for volunteers from students enrolled in psychology or health and safety courses. We excluded participants who had previously completed the same questionnaires in the authors’ class to avoid duplicate participants. The Ethics Committee of the Faculty of Arts and Science, Kyushu University, Japan approved this study.

### Questionnaires

#### Japanese version of the adult ADHD self-report scale (ASRS)

The ASRS is a self-reported questionnaire designed to screen for adult ADHD [20], and it consists of 18 items rated on a five-point scale (0 = never, 1 = rarely, 2 = sometimes, 3 = often, 4 = very often). We calculated the total score by summing all item values (from 0 to 72), with higher scores indicating more pronounced symptoms. Furthermore, we calculated two subscales: inattention (IA) and hyperactivity/impulsivity (H/I), according to previous reports [21, 22]. The 18 items were divided into two groups as follows— items related to IA (item numbers 1, 2, 3, 4, 7, 8, 9, 10, 11) and those related to H/I (item numbers 5, 6, 12, 13, 14, 15, 16, 17, 18). Thus, the total score of IA or H/I ranged from 0 to 36. Six of the questions (part A: items 1 to 6) are often used to diagnose adult ADHD, since these items are reported to be the most predictive of symptoms consistent with ADHD. Each item has a cut-off value (COV) of either 2 (sometimes) or 3 (often) and having four or more items in part A (ASRS-6) above the cut-off is a clinical sign of adult ADHD.

#### Japanese version of the autism-Spectrum quotient with 16 items (AQ16)

The AQ (Autism-Spectrum Quotient), developed by Baron-Cohen et al. [23], is a 50-item self-reported questionnaire that measures autistic traits. The AQ uses a four-point Likert-type scale including strongly agree, slightly agree, slightly disagree, and strongly disagree, with slightly or strongly agree responses each scoring 1 point, resulting in the possible total score ranging from 0 to 50. Although the AQ is widely used to measure autism spectrum tendencies in clinical research, the full 50-item version of the AQ is too lengthy to be included in comprehensive screenings for psychiatric disorders. Therefore, various research groups have developed shorter versions of the AQ as screening tools for autism spectrum disorder [24, 25]. To examine the extent of autistic traits in participants, we used the Japanese version of the Autism-Spectrum Quotient with 16 items (AQ16), which was designed to detect Asperger’s syndrome (AS) [26]. The AQ16 consists of 16 (Nos.7, 15, 18, 20, 24, 26, 27, 31, 32, 34, 35, 39, 41, 42, 45, 46) of the 50 items in the original version of the AQ [23]. According to previous research, a cut-off value of 12 on the AQ16 (score range: 0 to 16) indicates high sensitivity (0.80) and specificity (0.97) [26], and we therefore adopted the 12-point cut-off value for the present study.

#### Clutter image rating (CIR)

The clutter image rating [27] is a three-item picture scale that assesses levels of clutter by presenting a series of pictures of rooms in various stages of clutter. Participants are asked to match their level of clutter to one of nine pictures, with higher numbers indicating greater clutter. Summed scores range from 3 to 27 and the scores for three representative rooms (bedroom, kitchen, and living room) are averaged. The internal consistency across the three rooms was good (α = 0.855). A cut-off score of 4 or higher was used to indicate significant clutter tendencies that require clinical attention [27].

### Statistical analysis

Data were analyzed using IBM SPSS Statistics version 23.0 (IBM Corporation, Armonk, NY, USA). Chi-square or Fisher’s exact tests were used to compare the categorical variables. The Shapiro-Wilk test was employed to evaluate whether each set of measures was a normally distributed trait. Comparisons between men and women were performed using the Mann-Whitney *U* test. Data are shown as means (± SD) in Table 1. The relationships between pairs of variables were examined using Spearman’s correlation coefficient (Table 3). Statistical significance was set at *p* < 0.05.

**Table 1:** Sex differences in each measure

Measures	Total (mean ± SD)	Men (mean ± SD)	Women (mean ± SD)	*p* value (men vs women)
CIR				
Bedroom	1.86 ± 1.02	1.86 ± 1.06	1.83 ± 0.85	0.58
Kitchen	1.64 ± 0.88	1.67 ± 0.92	1.56 ± 0.74	0.25
Living room	1.96 ± 0.98	1.99 ± 1.03	1.88 ± 0.81	0.48
Composite	1.82 ± 0.85	1.83 ± 0.89	1.76 ± 0.68	0.71
ASRS				
Total score	24.9 ± 8.15	24.8 ± 7.72	25.1 ± 9.47	0.72
IA score	15.0 ± 4.56	14.9 ± 4.57	15.5 ± 5.70	0.33
H/I score	9.88 ± 4.43	9.96 ± 4.30	9.62 ± 4.81	0.14
AQ16				
Total score	5.88 ± 2.88	5.76 ± 2.85	6.24 ± 2.88	0.06

The *p*-values were examined with the Mann-Whitney *U*-test. *Abbreviations*: *CIR* Clutter Image Rating, *ASRS* Adult ADHD Self-Report Scale, *IA* inattention, *H/I* hyperactivity/impulsivity, *AQ16* Autism-Spectrum Quotient with 16 items

## Results

**Fig. 1:**
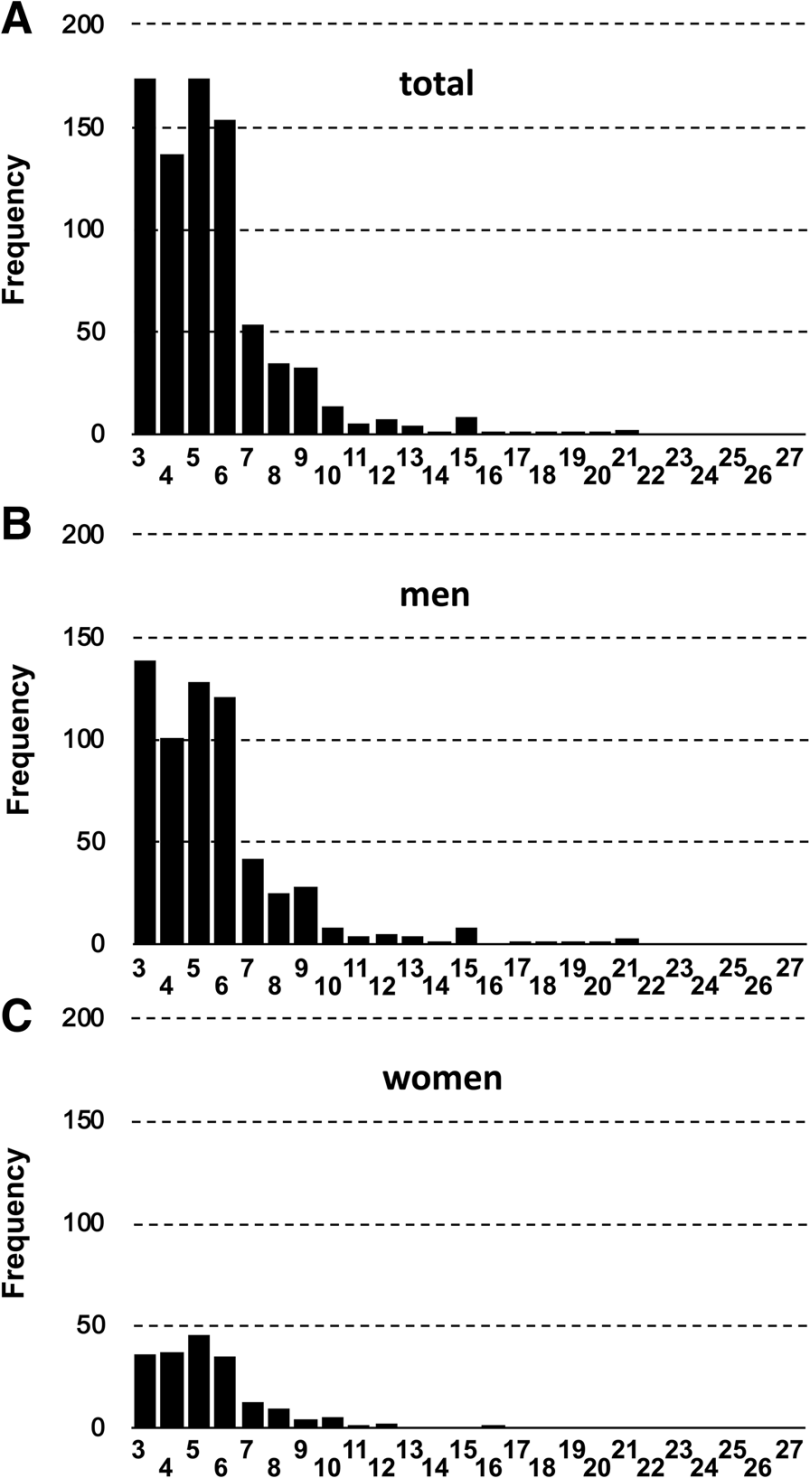
Distribution of CIR summed scores. **a** Total (*N* = 801), (**b**) Men (*N* = 616), and (**c**) Women (*N* = 185). The horizontal axis indicates the CIR summed score. The vertical axis indicates the number of participants (frequency) with each CIR score

In total, 801 students (616 men, 185 women) completed all of the questionnaires. Figure 1 displays the distribution of summed CIR scores. The mean participant age was 21.4 years (SD = 2.0 years). Among 801 participants, 27 (3.4%) exceeded the CIR cut-off value (95% confidence interval: 2.1–4.7%), including 24 (3.9%) of 616 men and 3 (1.6%) of 185 women. However, the Fisher’s exact test showed that there were no significant differences between men and women regarding hoarding behavior prevalence (*p* = 0.166). Table 1 shows comparisons between the participating men and women for each measure. The mean (± SD) CIR composite score was 1.82 (± 0.85), with no significant differences between men and women (men vs women; 1.83 ± 0.89 vs 1.76 ± 0.68). Although the men’s individual scores were slightly higher than the women’s, there were no statistically significant differences between men and women on the CIR subscale scores. Neither the ASRS nor the AQ16 showed statistically significant differences between men’s and women’s scores (Table 1).

Tables 2 and 3 illustrate the percentages of hoarding behavior comorbidities. To examine the relationships between hoarding behavior and developmental disorder traits, we divided the participants according to each questionnaire’s cut-off value as follows: HB (+), CIR (≥ 4); HB (−), CIR (< 4); ADHD (+), ASRS (part A ≥ 4); ADHD (−), ASRS (part A < 4); ASD (+), AQ16 (≥ 12); ASD (−), and AQ16 (< 12). First, we examined the proportion of participants with hoarding behavior and ADHD traits (Table 2). In the HB (−) group, 21.7% of participants were ADHD (+). In contrast, 40.7% of the HB (+) participants were also ADHD (+). In the ADHD (−) and ADHD (+) participants, the proportion of HB (+) was 2.6 and 6.1%, respectively. A chi-square test of independence revealed a significant interaction (χ2(1) = 5.45, *p* = 0.020). Next, we examined the proportion of participants with hoarding behavior and ASD traits (Table 3). In the HB (−) group, 4.1% of the participants were ASD (+), as were 7.4% of the HB (+) participants. In ASD (−) and ASD (+) participants, the proportion of HB (+) was 3.3 and 5.9%, respectively. However, the Fisher’s exact test showed no significant between-group differences (*p* = 0.320).

**Table 2:** Cut-off values and number of subjects in each group (CIR and ASRS)

	CIR COV (≥ 4)		HB ratio (%)	Total	*p*-value
	Not exceed: HB(−)	Exceeded: HB(+)			
ASRS COV (part A ≥ 4)					0.020*
Not exceed: ADHD (−)	606	16	2.6	622	
Exceeded: ADHD (+)	168	11	6.1	179	
ADHD ratio (%)	21.7	40.7		22.3	
Total	774	27	3.4	801	

*p < 0.05 indicates statistical significance. The *p*-values were calculated from chi-square tests for either group distribution. *Abbreviations*: *COV* cut-off value, *HB* hoarding behavior, *CIR* Clutter Image Rating, *ASRS* adult ADHD Self-Report Scale

**Table 3:** Cut-off values and number of subjects in each group (CIR and AQ16)

	CIR COV (≥ 4)		HB ratio (%)	Total	*p*-value
	Not exceed: HB(−)	Exceeded: HB(+)			
AQ16 COV (≥ 12)					0.320
Not exceed: ASD (−)	742	25	3.3	767	
Exceeded: ASD (+)	32	2	5.9	34	
ASD ratio (%)	4.1	7.4		4.2	
Total	774	27	3.4	801	

*p* < 0.05 indicates statistical significance. The *p*-values were calculated from Fisher’s exact tests for either group distribution. *Abbreviations*: *COV* cut-off value, *HB* hoarding behavior, *CIR* Clutter Imager Rating, *AQ16* Autism-Spectrum Quotient with 16 items

Next, we examined correlations among all the study variables. The results are shown in Table 4. We found significant correlations among all scores on the CIR, ASRS, and AQ16. The intercorrelations among the three CIR rooms were moderate to strong (bedroom vs living room, *r* = 0.663; bedroom vs kitchen, *r* = 0.422; living room vs kitchen, *r* = 0.539). The intercorrelation between the two subscales of the ASRS was moderate (IA vs H/I, *r* = 0.499). The correlation among CIR composite, ASRS total, and AQ16 total scores was weak or moderate (CIR composite vs ASRS total, *r* = 0.250; CIR composite vs AQ16 total, *r* = 0.194; ASRS vs AQ16 total, *r* = 0.413). The correlations between the CIR composite score and the ASRS subscales were weak or very weak (CIR composite vs ASRS IA, *r* = 0.283; CIR composite vs ASRS H/I, *r* = 0.147).

**Table 4:** Correlations among the CIR, ASRS and AQ16 scores

	CIR				ASRA			AQ16
	Bed	Kitchen	Living room	Composite	Total	IA	H/I	Total
CIR								
Bedroom	–							
Kitchen	.422^a^	–						
Living room	.633^a^	.539^a^	–					
Composite	.839^a^	.746^a^	.883^a^	–				
ASRS								
Total	.232^a^	.198^a^	.203^a^	.250^a^	–			
IA	.266^a^	.207^a^	.232^a^	.283^a^	.870^a^	–		
H/I	.127^a^	.133^a^	.127^a^	.147^a^	.846^a^	.499^a^	–	
AQ16								
Total	.183^a^	.136^a^	.157^a^	.194^a^	.413^a^	.434^a^	.284^a^	–

^a^Correlation is significant at < 0.001. The *p*-values were calculated from Spearman’s correlation coefficient. *Abbreviations*: *CIR* Clutter Image Rating, *ASRS* adult ADHD Self-Report Scale, *IA* inattention, *H/I* hyperactivity/impulsivity, *AQ16* Autism-Spectrum Quotient with 16 items

## Discussion

This study is the first large-scale survey to identify the relationships between hoarding behavior and developmental disorders in Japanese young adults. Our results gave a 3.4% prevalence rate for hoarding behavior among these university students. Moreover, we found that the participants with hoarding behavior had a significantly higher percentage of ADHD traits than participants without hoarding behavior (HB(+) vs HB(−), 40.7% vs 21.7%). Furthermore, we found statistically significant correlations among CIR, ASRS, and AQ16 (CIR vs ASRS, *r* = 0.25; CIR vs AQ16, *r* = 0.194; ASRS vs AQ16, *r* = 0.413). Finally, we found that the sub-scales of ASRS (inattentive symptoms and hyperactivity/impulsivity symptoms) have a statistically significant correlation with hoarding behavior (CIR vs IA, *r* = 0.283; CIR vs H/I, *r* = 0.147).

There are several epidemiological studies that examine the prevalence of hoarding behavior in the general population [4, 7, 28]; however, few studies have surveyed the prevalence of hoarding behavior in young adults. Two recent studies reported about a 1% prevalence of hoarding disorder in young adults [29, 30]. In our study, there was a 3.4% prevalence rate for hoarding behavior among university students, suggesting that our result was three times greater than the rates reported in these two previous studies. However, our research focused on university students whereas these other studies were population-based research and thus included various socio-economic indicators (e.g., occupation, economic status, and race). Furthermore, the previous studies utilized the Hoarding Rating Scale-Self Report to evaluate hoarding symptoms, in contrast to the CIR used in the present study. Thus, the difference in our hoarding behavior results in comparison to previous findings might be explained by these variations in study participants and in the evaluation methods. In addition, previous studies regarding sex differences in the prevalence of hoarding behavior have not been consistent. Many studies have reported no difference between men and women, while others have reported a higher prevalence of hoarding in men [4, 7, 31, 32]. In the present study, men had a higher prevalence of hoarding behavior than women (3.9 and 1.6%, respectively); however, this difference was not statistically significant. Since sex-based differences in hoarding behavior may be influenced by sampling differences or cultural variations [32], further studies are needed to investigate this inconsistency.

Over the past 15 years, evidence has accumulated to support findings of high levels of comorbidity between hoarding behavior and ADHD. For example, Frost et al. reported that 30% of people with hoarding disorder met the criteria of inattentive ADHD [2]. In addition, in a small sample study, Hartl et al. showed that 32% of the hoarding participants were also diagnosed as ADHD [33]. In the current study, more than 40% of the university students with hoarding behavior exceeded the ASRS cut-off value, supporting previous studies’ reports of high comorbidity between hoarding disorder and ADHD. Previous studies have also reported that some ADHD symptoms are related to hoarding behavior. Fullana et al. investigated the association between retrospectively reported ADHD symptoms in childhood and lifetime hoarding symptoms in a representative sample from the general population, and they found that lifetime hoarding symptoms were more common among participants with childhood ADHD symptoms than those without ADHD symptoms (8.9% vs 2.7%) [34]. In addition, two studies have suggested a more specific relation between ADHD symptoms and hoarding, with inattention but not hyperactivity being associated with hoarding in adult cases [2, 35]. Frost et al. studied such comorbidity in 217 participants with hoarding behavior, and they found that inattentive ADHD was diagnosed in 28% of their participants with hoarding disorder; in contrast, hyperactive ADHD was diagnosed in 13.7% of their participants [2]. Tolin et al. examined the relationship between the core features of hoarding, OCD symptoms, and ADHD symptoms, and clarified that the inattentive (but not hyperactive/impulsive) symptoms of ADHD significantly predicted the severity of clutter, difficulty discarding items [35]. In the present study, we found that CIR composite scores had a stronger correlation with ASRS inattentive scores than with hyperactivity/impulsivity scores. Hence, our results align with previous reports that inattentive symptoms are more closely related to hoarding behavior than are hyperactivity/impulsivity symptoms.

There is also accumulating evidence to explain the high comorbidity of hoarding behavior and traits of ADHD. For example, some neuropsychological research has shown that patients with hoarding behavior have problems with attention and decision-making [36], which are also observed in patients with ADHD [37]. Lawrence et al. found that hoarding symptoms were associated with specific decision-making impairments on the Iowa Gambling Task and that these deficits were related to hoarding symptom severity [38]. Hoarders’ impulsive acquisition and difficulty making decisions about discarding and organizing possessions may be derived from similar information-processing difficulties [39]. In a genetic study, exploratory factor analyses revealed that hoarding behavior was associated with ADHD symptoms in Tourette syndrome families [40]. This finding suggests the presence of genetic abnormalities common to hoarding behavior and ADHD. Furthermore, one clinical study showed that atomoxetine, which is a medication for ADHD, was effective for hoarding disorder, implying that these two disorders may share a common neurochemical dysfunction [41]. Thus, both hoarding behavior and ADHD may have common pathological pathways, resulting in their high rates of comorbidity.

Given the comorbidity and positive correlation between hoarding behavior and ADHD traits, our results imply the importance of assessing for hoarding behavior in young adults with ADHD. Hoarding behavior often emerges before the age of 20; however, hoarding symptoms per se are not typically severe during childhood and adolescence [42]. Hoarding behavior may be unremarkable during childhood because parents can prevent clutter and children do not have enough money to acquire items [43]. Since young adults in Japan, especially university students, often live alone after graduating from high school, hoarding behavior may only emerge after they leave their parents. Given that hoarding symptoms appear to become chronic and tend to worsen over a person’s lifetime, it is important to detect and treat hoarding behavior as soon as the symptoms begin [44]. Meta-analyses have indicated that cognitive-behavioral therapy (CBT) is effective for hoarding symptoms, including difficulty discarding, clutter, and acquiring, and that CBT intervention at a younger age is predictive of significantly better outcomes in overall hoarding severity and acquiring [45]. Likewise, it has been proposed that early detection and treatment may be effective for reducing the likelihood of persistent ADHD [46]. For example, both pharmacological and psychological intervention in children and adolescents are effective ADHD treatments [47]. Furthermore, there is evidence to suggest that early ADHD treatment has a protective effect on future development of depression, bipolar disorder, and substance abuse [48]. Taken together, early intervention for hoarding behavior and ADHD appears to be beneficial for young adults, and interventions for hoarding behavior may improve quality of life, including physical and mental health, school performance, and social adaptation in university students with ADHD.

In the present study, we found that the prevalence of hoarding behavior among participants with ASD (+) (AQ16 ≥ 12) was lower than in the ADHD (+) group (7.4% vs 40.7%, respectively). In addition, correlation analyses indicated that there was a statistically significant but weak correlation between AQ16 score and CIR scores (Table 4). However, unlike our results, several previous reports have shown that hoarding was significantly related with ASD. Ruta et al. investigated obsessive-compulsive traits in children and adolescents with AS using the Children’s Yale-Brown Obsessive-Compulsive Scale (CY-BOCS), and they found that the AS group presented significantly higher frequencies of hoarding behavior compared to the control group (44% vs 9%, respectively) [49]. Scahill et al. examined the repetitive behavior of ASD using the modified CY-BOCS and reported that 24.3% of their participants with ASD (aged 4–17 years) exhibited hoarding behavior [50]. Furthermore, La Buissonniere-Ariza et al. recently reported that 34% of children with ASD presented at least moderate levels of hoarding behavior, with 7% presenting severe to extreme levels of hoarding [51]. The major difference between our results and those of previous studies centers on whether or not the participants were clinically diagnosed with ASD. Our study was based on questionnaire responses, thus only non-clinical cases were included. However, previous studies examined participants clinically diagnosed as ASD. Moreover, participants in our study were university students, whereas previous studies focused on children or adolescents. These differences in experimental design may explain the low prevalence of hoarding behavior among ASD (+) participants in our study.

In the present study, we examined hoarding behavior from the viewpoint of developmental disorders; however, it has been suggested that hoarding behavior has relevance with other psychiatric disorders other than OCD spectrum disorders. For example, a previous study reported that 7.7% of patients with major depression and 7.4% of patients with anxiety disorders exceeded the cut-off for a diagnosis of hoarding disorder [52]. Significant hoarding symptoms were also found among patients seeking treatment for anxiety disorders, especially those diagnosed with generalized anxiety disorder (24%) and with social phobia (11%) [53]. In the same study, it was also identified that hoarding symptoms were positively correlated with trait anxiety and depressive symptoms [53]. Because it is difficult to attain clinical diagnoses, there are only a few large-scale studies in Japan that have examined the exact prevalence of depression/anxiety disorders among university students. Instead, there are many self-reported assessment studies, given the ease and availability of self-reported assessments. For example, in a study that utilized the Liebowitz Social Anxiety Scale, a screening test for social anxiety disorder, 10.3% of 673 university students exceeded the cut-off value for social anxiety disorder [54]. In addition, another study utilized the diagnostic tool for depression, the Patient Health Questionnaire-9, to report that 8% of 1421 university students have had either a major depressive episode or another depressive episode [55]. Thus, since anxiety and depressive symptoms are prevalent among Japanese university students, it is possible that these symptoms affect the onset or severity of hoarding behavior. Therefore, in addition to developmental disorders, it will be important for future research to examine hoarding behavior in young adults from the standpoint of the anxiety-depression spectrum.

The current study has some limitations. First, all participants were recruited from a single University. In Kyushu University, about 70% of the students are male, suggesting that our results cannot be generalized to all young adults in Japan. Second, the present study used the original version of the CIR (developed by Frost et al.) [27]. Since this study was conducted in Japan, it would have been appropriate to use the Japanese version of the CIR; however, the reliability and validity of the Japanese version of the CIR have not yet been confirmed. Since hoarding behavior is likely to be affected by a person’s social environment, it is necessary to develop a reliable and valid Japanese version of the CIR to more accurately screen hoarding behavior in Japan. Third, we relied on self-reported assessments to diagnose hoarding behavior, ASD, and ADHD. In general, a clinical diagnosis is determined by a psychiatric interview, according to operational diagnostic criteria. Therefore, a clinical evaluation via a structured interview would be required for accurate diagnoses of hoarding disorder, ASD, and ADHD. Particularly, hoarding behavior is often observed in other psychiatric disorders such as eating disorders, major depression, and psychotic disorders [52, 56], and it is important to examine the comorbid disorders to understand the pathology of hoarding.

## Conclusion

In conclusion, our study demonstrated that there is a high prevalence of ADHD traits in university students with hoarding behavior. Moreover, we found that hoarding behavior was more strongly correlated with inattentive symptoms than with hyperactivity/impulsivity. Our results may help elucidate the common pathophysiology underlying hoarding behavior and ADHD in young adults. Further studies are needed to develop effective interventions for young adults with hoarding behavior from the standpoint of developmental disorders.

## Data Availability

The data are available upon reasonable request.

## Abbreviations

ADHD: Attention deficit hyperactivity disorder
AQ16: Autism-Spectrum Quotient with 16 items
AS: Asperger’s syndrome
ASD: Autism spectrum disorder
ASRS: Adult ADHD Self-Report Scale
CIR: Clutter Image Rating
COV: Cut-off value
H/I: Hyperactivity/impulsivity
HB: Hoarding behavior
IA: Inattention
OCD: Obsessive-compulsive disorder
